# Age and mini-mental state examination score can predict poor-quality spirometry in the elderly: a cross-sectional study

**DOI:** 10.6061/clinics/2018/e374

**Published:** 2018-09-24

**Authors:** Rodrigo Santos de Queiroz, Luciano Magno de Almeida Faria, José Ailton Oliveira Carneiro, Raildo da Silva Coqueiro, Marcos Henrique Fernandes

**Affiliations:** Departamento de Saude 1, Campus de Jequie, Universidade Estadual do Sudoeste da Bahia, Jequie, BA, BR

**Keywords:** Respiratory Function Tests, Spirometry, Quality Control, Aged, Mild Cognitive Impairment

## Abstract

**OBJECTIVES:**

The goal was to identify predictors of poor-quality spirometry in community-dwelling older adults and their respective cutoffs.

**METHODS:**

This was a cross-sectional population-based study involving 245 elderly subjects (age≥60 years). The spirometric data were categorized as good or poor quality, and cognitive status was assessed using an adapted version (scaled to have a maximum of 19 points) of the Mini-Mental State Examination. Multivariate analysis was used to assess the association between poor-quality spirometry and sociodemographic, behavioral and health characteristics. The best cutoff points for predicting poor-quality spirometry were evaluated by the receiver operating characteristic curve.

**RESULTS:**

In this population, 61 (24.9%) subjects with poor-quality spirometry were identified. After multiple logistic regression analysis, only age and Mini-Mental State Examination score were still associated with poor-quality spirometry (*p*≤0.05). The cutoff for the Mini-Mental State Examination score was 15 points, with an area under the receiver operating characteristic curve of 0.628 (*p*=0.0017), sensitivity of 74.5% and specificity of 49.5%; for age, the cutoff was 78 years, with an area under the receiver operating characteristic curve of 0.718 (*p*=0.0001), sensitivity of 57.4% and specificity of 79.9%.

**CONCLUSION:**

Age and Mini-Mental State Examination score together are good predictors of poor-quality spirometry and can contribute to the screening of community-dwelling older adults unable to meet the minimum quality criteria for a spirometric test.

## INTRODUCTION

Spirometry is the most important test for evaluating respiratory function; however, the elderly have high rates (12% to 17.4%) of inability to meet the quality criteria 1-3 because of the maximal, successive, sustained and coordinated expiratory efforts required to perform spirometric maneuvers accurately 4.

In many geriatric practices, spirometric evaluation is not widely used; thus, experience with elderly patients, especially community-dwelling elderly subjects, is still limited. Therefore, good predictors of poor-quality spirometry have not been identified for this population.

Identifying predictors of poor-quality spirometry in community-dwelling elderly subjects can facilitate more specific screening of people who need special attention or need more time to learn about the test and acquaint themselves with the controls and the techniques of the test; this screening could also reduce the suffering that occurs with repeated unsuccessful expiratory attempts, alerting clinicians to the need for other means of diagnosing pulmonary function 2. Thus, the aim of this study was to identify predictors of poor-quality spirometry in community-dwelling elderly people and the cutoffs for these predictors.

## METHODS

This was an analytical, cross-sectional study that analyzed data from a home-based epidemiological research called “*Nutritional status, risk behaviors and health conditions of elderly in Lafaiete Coutinho, BA, Brazil.*” The study protocol was approved by the Research Ethics Committee of the State University of Southwest Bahia (No. 491.661/2014), and all participants were informed about the procedures and signed an informed consent form.

The city of Lafaiete Coutinho had 3901 inhabitants in 2010, with an estimated population of 3975 in 2017 5. A census of elderly people (age≥60 years) was performed for all residents in the urban zone and enrolled in the Family Health Strategy, which covered the whole population of the city. Of the 331 elderly people who comprised the study population, three refused to participate, and 10 were not found in their homes even after three visits on different days and at different times. Ultimately, 318 (96%) elderly people participated in the home interviews.

The data were collected in two stages: The first was a home interview using a form based on the questionnaire used in the survey on Health, Well-being, and Aging – SABE 6. The second stage was performed in the two health units of the city, where anthropometric measurements, motor tests and spirometric maneuvers were carried out.

**Figure 1:**
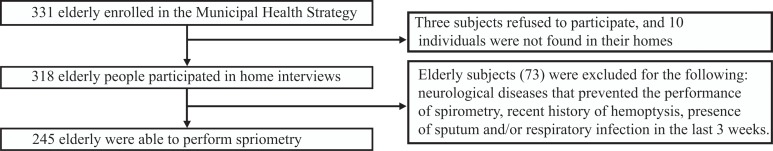
Flowchart of the study participants.

A screening was performed to verify suitability to perform spirometry. Seventy-three (23.0%) were excluded from the study. The exclusion criteria were as follows: neurological disorders that made it impossible to carry out the evaluation and conditions that contraindicate the performance of spirometry (a recent history of hemoptysis or the presence of sputum and respiratory infection in the past 3 weeks). Spirometry maneuvers were performed in 245 (77.0%) elderly people (Figure 1).

### Spirometry

Spirometry was performed using a MicroLab™ spirometer (Care Fusion, USA) connected to a computer. The spirometry software provided real-time feedback on the flow volume curves and an automated quality analysis that met all the criteria of the American Thoracic Society and European Respiratory Society 3. The maneuvers were repeated with a minimum of three and no more than eight forced expiratory efforts. All tests were performed by a single researcher. The quality of the spirometry was further confirmed by an independent researcher.

To perform the analyses, the spirometry results were divided into two categories. 1) Good-quality spirometry: tests that met the minimum acceptance/repeatability criteria (i.e., a minimum of two acceptable and reproducible expiratory efforts, where the difference between the two largest values of forced vital capacity (FVC) and the two largest values of forced expiratory volume in one second (FEV_1_) were ≤0.200 L) and values of <15% for the variation in peak expiratory flow (PEF); 2) poor-quality spirometry: acceptable expiratory maneuvers, with values of FVC and/or FEV_1_ that showed variations greater than 0.200 L 7.

### Sociodemographic, behavioral and health characteristics

Information was collected regarding age, gender, ability to read and write a message (categorized as yes and no), and tobacco use (never smoked, former smokers and currently smoke). The number of chronic diseases (none, one, and two or more), such as hypertension, diabetes, cancer (except skintumors), chronic lung disease, heart diseases, circulatory diseases, rheumatic diseases and osteoporosis, was collected through self-report. Weight and height were measured and used to calculate body mass index (BMI).

The degree of dyspnea on exertion was determined using the adapted version of the questionnaire of the American Thoracic Society Division of Lung Disease Questionnaire (ATS-DLD) dichotomized into mild (degrees 0, 1 and 2) and severe (degrees 3 and 4) degrees of dyspnea 8. Cognitive status was assessed using the adapted version (maximum score of 19 points) 9 of the Mini-Mental State Examination (MMSE) 10. Depressive symptoms were assessed using the Geriatric Depression Scale (GDS) in the abbreviated form of 15 items 11, categorized into normal (<6 points) and risk of depressive symptoms (≥6 points). Functional capacity was considered in a hierarchical way 12 and divided into three categories: independent, dependent on the instrumental activities of daily living (IADL) 13 and dependent on the basic activities of daily living (BADL) 14 and IADL. Handgrip strength (kg) was measured using a hydraulic dynamometer (Saehan Corporation SH5001, Korea). For assessing the physical activity level, the International Physical Activity Questionnaire (IPAQ), long version 15, was used, with categorization (from moderate or vigorous physical activity level per week) into active (≥150 minutes) and insufficiently active (<150 minutes) 16.

### Statistical analysis

Descriptive analyses were performed for all variables (categorical: absolute and relative frequency; continuous: mean, median, interquartile range and standard deviation). The association between poor-quality spirometry and sociodemographic, behavioral and health characteristics was tested initially through binary logistic regression (crude association), and then, the analysis was adjusted for variables with *p*≤0.20. The adjusted analysis was performed through multiple logistic regression (input method: stepwise forward). In all stages of analysis, the odds ratios were estimated with a 95% confidence interval. After the adjustment, the variables with *p*≤0.05 remained. The best cutoffs for predicting poor-quality spirometry were evaluated by the parameters provided by the receiver operating characteristic curve (ROC), the area under the ROC curve (AUC), sensitivity and specificity. Analyses were performed using Statistical Package for the Social Sciences (SPSS) for Windows - version 21.0 (IBM Corp, USA) and MedCalc for Windows - version 9.1.0.1 (MedCalc Software, Belgium).

## RESULTS

The study population consisted of 132 women (53.9%) and 113 men (46.1%), with a mean age of 73.3±8.6 years; the age range was 60 to 95 years, and 61 (24.9%) of the elderly were 80 years of age or older. The MMSE was completed by 237 (96.7%) elderly subjects. Ninety elderly subjects (38.0%) presented low scores (MMSE <13), and of these, only 59 (32.4%) presented good-quality spirometry. Sixty-one (24.9%) examples of poor-quality spirometry were identified.

Table 1 shows the results of the bivariate analysis of the association between poor-quality spirometry and the sociodemographic, behavioral and health characteristics of the elderly participants of the study. Age, BMI, ability to read and write a message, MMSE score, GDS-15, handgrip strength and activity physical level had *p*≤0.20.

**Figure 2:**
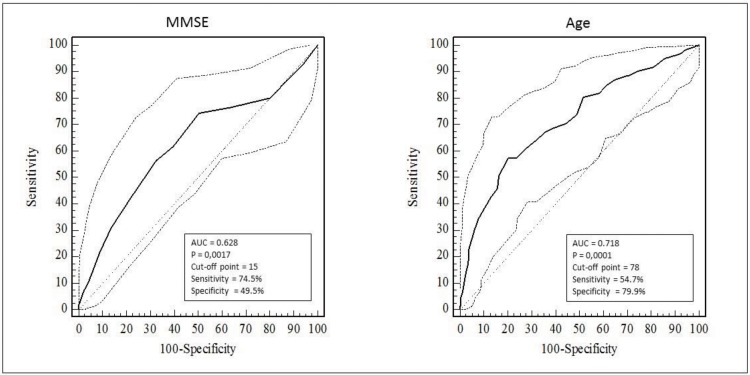
Area under the ROC curve of MMSE and age as predictors of poor quality spirometry in community-dwelling elderly.AUC, Area under the ROC curve; MMSE, Adapted version of the Mini-Mental State Examination.

After the multiple logistic regression, BMI, ability to read and write a message, GDS-15, handgrip strength and activity physical did not remain in the model. Only age and MMSE score maintained an association (*p*≤0.05) with poor-quality spirometry (Table 2). The AUC (Figure 2) indicated that MMSE score and age had the potential to distinguish the capacity of an elderly person to perform spirometry properly; the MMSE score had higher sensitivity, and age had greater specificity.

## DISCUSSION

The frequency of poor-quality spirometry was high among the evaluated elderly subjects and higher than that in other studies in the area 1-3,17. Among the variables studied, only age and MMSE score were factors that were associated with poor-quality spirometry. The cutoffs for age and MMSE score were identified to be 78 years and 15 points, respectively, and were able to predict poor-quality spirometry in the elderly. Age had high specificity, while MMSE score showed high sensitivity.

To execute the spirometric test properly, elderly subjects need to perform deep breaths and explosive and sustained expirations. Therefore, the forced expiratory maneuvers of spirometry may represent a demanding physical activity with which most elderly people are not familiar, resulting in a higher occurrence of poor-quality spirometry 1,3,17,18. This situation occurs because even with healthy aging, some degree of physiological and physical performance impairment is expected, especially in people of advanced age 2,19,20.

With advancing age, as well as physical decline, a cognitive decline occurs 20. Previous studies reported a significant association between cognitive impairment (based on the MMSE score) and poor-quality spirometry in elderly outpatients, hospitalized patients, institutionalized patients and respiratory impairment patients 1,2,18-21, but this association was not found in community-dwelling elderly people 3.

It has been suggested that MMSE <13 denotes cognitive impairment 22. The percentage of subjects with MMSE <13 was high in the study, possibly due to the low educational level, inferred from the ability to read and write a message (Table 1). In addition, there was a high prevalence of elderly individuals aged 80 years or older. The literature reports that younger age and higher education are associated with a better MMSE score 23.

The strengths of this study are that it is a population census, uses a single evaluator trained in spirometry, and has independent evaluation of the acceptability and reproducibility of the results in accordance with specific international guidelines for pulmonary function tests. The cutoffs for age and MMSE score can be used as objective parameters and guidance for stratification of the groups most likely to fail in performing the spirometry test.

One of the limitations of this study was the great loss of participants who did not meet the clinical criteria for inclusion at the time of collection. Additionally, there was no medical diagnosis of respiratory diseases, such as chronic obstructive pulmonary disease; therefore, this information was obtained through self-report.

In addition to screening the elderly for tests that do not require good cognitive status to evaluate lung function, this study may encourage a discussion that promotes specific criteria for the acceptance and reproducibility of spirometric maneuvers in elderly people over 78 years of age with cognitive impairment.

The results of this study indicated that age and impaired cognitive status (assessed by the MMSE) are good predictors for screening elderly people likely to produce poor-quality spirometry. The use of these two predictors together can contribute to screening elderly subjects unable to meet the minimum quality criteria for spirometry.

## AUTHOR CONTRIBUTIONS

Queiroz RS conception or design of the work; Acquisition (collection), analysis and interpretation of data for the work; Drafting the manuscript or revising it critically for important intellectual content; Final approval of the version to be published; Agreement to be accountable for all aspects of the work by ensuring that questions related to the accuracy or integrity of any part of the work are appropriately investigated and resolved. Faria LM acquisition (collection) of data; Drafting the manuscript or revising it critically for important intellectual content. Carneiro JA acquisition (collection), analysis and interpretation of data for the work; Drafting the manuscript or revising it critically for important intellectual content. Coqueiro RS analysis and interpretation of data for the work; Drafting the manuscript or revising it critically for important intellectual content. Fernandes MH conception or design of the work; Acquisition (collection), analysis and interpretation of data for the work; Drafting the manuscript or revising it critically for important intellectual content; Final approval of the version to be published.

## Figures and Tables

**Table 1:** Characteristics of the study population and association with spirometry quality.

Characteristic	Spirometry quality		ORCrude (95% CI)	*p*-value
	Good (*n*=184)	Poor (*n*=61)		
Age (years), mean ± s.d.	71.5±7.8	77.9±8.7	1.10 (1.06-1.14)	<0.001
Sex (men), *n* (%)	86 (76.1)	27 (23.9)	0.90 (050-1.62)	0.737
BMI (kg/m2), mean ± s.d.	25.6±4.9	24.6±3.2	0.95 (0.89-1.02)	0.198
Ability to read and write a message, *n* (%)				
Yes	77 (82.8)	16 (17.2)	1	
No	104 (70.3)	44 (29.7)	2.03 (1.07-3.87)	0.030
Smoking status, *n* (%)				
Never	76 (72.4)	29 (27.6)	1	
Previous	86 (78.9)	23 (21.1)	0.70 (0.37-1.31)	0.268
Current	18 (72.0)	7 (28.0)	1.01 (0.38-2.69)	0.969
Number of chronic diseases, *n* (%)				
No	23 (69.7)	10 (30.3)	1	
One	62 (72.1)	24 (27.9)	0.89 (0.37-2.14)	0.796
Two or more	88 (77.2)	26 (22.8)	0.68 (0.28-1.60)	0.380
ATS-DLD score, *n* (%)				
Mild (0-2)	130 (78.8)	35 (21.2)	1	
Severe (3-4)	42 (73.7)	15 (26.3.4)	1.32 (0.66-2.66)	0.427
MMSE score, median (IQR)	15 (13-17)	13 (11-16)	0.85 (0.77-0.94)	0.002
GDS-15 categories, *n* (%)				
Normal (<6)	160 (77.7)	46 (22.3)	1	
Risk for depression (≥6)	22 (61.1)	14 (38.9)	2.21 (1.05-4.66)	0.037
Functional capacity, *n* (%)				
Independent	115 (77.2)	34 (22.8)		
Dependent for IADL	41 (71.9)	16 (28.1)	1.32 (0.66-2.63)	0.432
Dependent for BADL and IADL	26 (72.2)	10 (27.8)	1.30 (0.57-2.96)	0.531
Handgrip strength (kg), median (IQR)	24 (19-30)	22 (17-26)	0.94 (0.90-0.98)	0.011
IPAQ categories, *n* (%)				
≥150 min/week	137 (80.1)	34 (19,9)	1	
<150 min/week	47 (63.5)	27 (36.5)	2.31 (1.26-4.23)	0.006

Abbreviations: OR, Odds ratio; CI, Confidence interval; IQR, Interquartile range; BMI, Body mass index; ATS-DLD, Adapted American Thoracic Society Division of Lung Disease Questionnaire; MMSE, Adapted Mini-Mental State Examination; Adapted GDS-15, 15-item Geriatric Depression Scale; IADL, Instrumental activities of daily living; BADL, Basic activities of daily living; IPAQ, International Physical Activity Questionnaire.

**Table 2:** Multivariable logistic regression analysis of the association of age and MMSE score with spirometry quality.

Variables	Spirometry quality	
	OR_adjusted_ (95% CI)	*p*-value
Age (years)	1.089 (1.04-1.13)	<0.001
MMSE score	0.89 (0.80-0.99)	0.035

Abbreviation: MMSE, Adapted version of the Mini-Mental State Examination.
